# Meta-analysis of Montreal cognitive assessment diagnostic accuracy in amnestic mild cognitive impairment

**DOI:** 10.3389/fpsyg.2024.1369766

**Published:** 2024-02-13

**Authors:** Michael Malek-Ahmadi, Nia Nikkhahmanesh

**Affiliations:** ^1^Banner Alzheimer’s Institute, Phoenix, AZ, United States; ^2^College of Medicine, University of Arizona, Phoenix, AZ, United States

**Keywords:** cognitive screen, mild cognitive impairment, cognitively unimpaired, diagnostic accuracy, cutoff score

## Abstract

**Background:**

The Montreal Cognitive Assessment (MoCA) is one of the most widely-used cognitive screening instruments and has been translated into several different languages and dialects. Although the original validation study suggested to use a cutoff of ≤26, subsequent studies have shown that lower cutoff values may yield fewer false-positive indications of cognitive impairment. The aim of this study was to summarize the diagnostic accuracy and mean difference of the MoCA when comparing cognitively unimpaired (CU) older adults to those with amnestic mild cognitive impairment (aMCI).

**Methods:**

PubMed and EMBASE databases were searched from inception to 22 February 2022. Meta-analyses for area under the curve (AUC) and standardized mean difference (SMD) values were performed.

**Results:**

Fifty-five observational studies that included 17,343 CU and 8,413 aMCI subjects were selected for inclusion. Thirty-nine studies were used in the AUC analysis while 44 were used in the SMD analysis. The overall AUC value was 0.84 (95% CI: 0.81, 0.87) indicating good diagnostic accuracy and a large effect size was noted for the SMD analysis (Hedge’s *g* = 1.49, 95% CI: 1.33, 1.64). Both analyses had high levels of between-study heterogeneity. The median cutoff score for identifying aMCI was <24.

**Discussion and conclusion:**

The MoCA has good diagnostic accuracy for detecting aMCI across several different languages. The findings of this meta-analysis also support the use of 24 as the optimal cutoff when the MoCA is used to screen for suspected cognitive impairment.

## Introduction

Amnestic mild cognitive impairment (aMCI) due to Alzheimer’s disease (AD) is a syndrome that is associated with future progression to clinical AD. While not all individuals with aMCI progress to AD, they are thought to be at the highest risk of progression and this classification is often referred to as “MCI due to AD” (Albert et al., 2011; Sperling et al., 2011). The diagnostic criteria for aMCI have remained largely the same since their initial publication (Petersen et al., 1999) and require that an individual’s episodic memory performance fall at least 1.5 standard deviations below what would be expected for their age and education level and is accompanied by a self-reported or collateral-reported complaint of cognitive decline. However, the aMCI diagnosis is made only after an extensive neuropsychological examination which prevents the diagnosis from being made in general practice settings where cognitive screening measures are often used to determine if an individual requires a more comprehensive cognitive assessment (Townley et al., 2019). Further refinements to the aMCI diagnostic criteria include the differentiation of those whose impairments are only in the memory domain (single domain) versus those who are impaired in memory and another cognitive domain (multiple domain) (Petersen and Negash, 2008). These classifications also apply for cases where the memory domain is not impaired (non-amnestic MCI), but other domains are (Petersen and Negash, 2008).

For several decades the Mini-Mental State Exam (MMSE) (Folstein et al., 1975) has been the most ubiquitous cognitive screening instrument, however the Montreal Cognitive Assessment (MoCA) (Nasreddine et al., 2005) is now among the most widely-used assessments for cognitive screening in general practice settings. Recent evidence indicates that the MoCA is superior to the MMSE in its ability to differentiate aMCI from normal cognition (Pinto et al., 2019a) as many individuals with aMCI often obtain normal scores on the MMSE (26–30) despite collateral reports of significant cognitive decline. The initial validation study of the MoCA recommended the same cutoff score as the MMSE (<26), however subsequent studies have indicated this cutoff may be too stringent and result in false positive indications of possible cognitive impairment (Wong et al., 2015; Carson et al., 2018; Ilardi et al., 2023).

To date, there has not been an extensive review and quantitative analysis of the MoCA’s diagnostic accuracy for aMCI. Given that the MoCA has been translated into many different languages and dialects it is important to understand how consistent its diagnostic accuracy is across its various translations. The aims of this meta-analysis are to characterize the MoCA’s diagnostic accuracy for aMCI and to characterize its relative effect size for mean differences between cognitively unimpaired (CU) older adults and those with aMCI using a large sample of published observational studies that cover a wide array of the languages that the MoCA has been translated in.

## Methods

### Inclusion criteria

**Figure 1:**
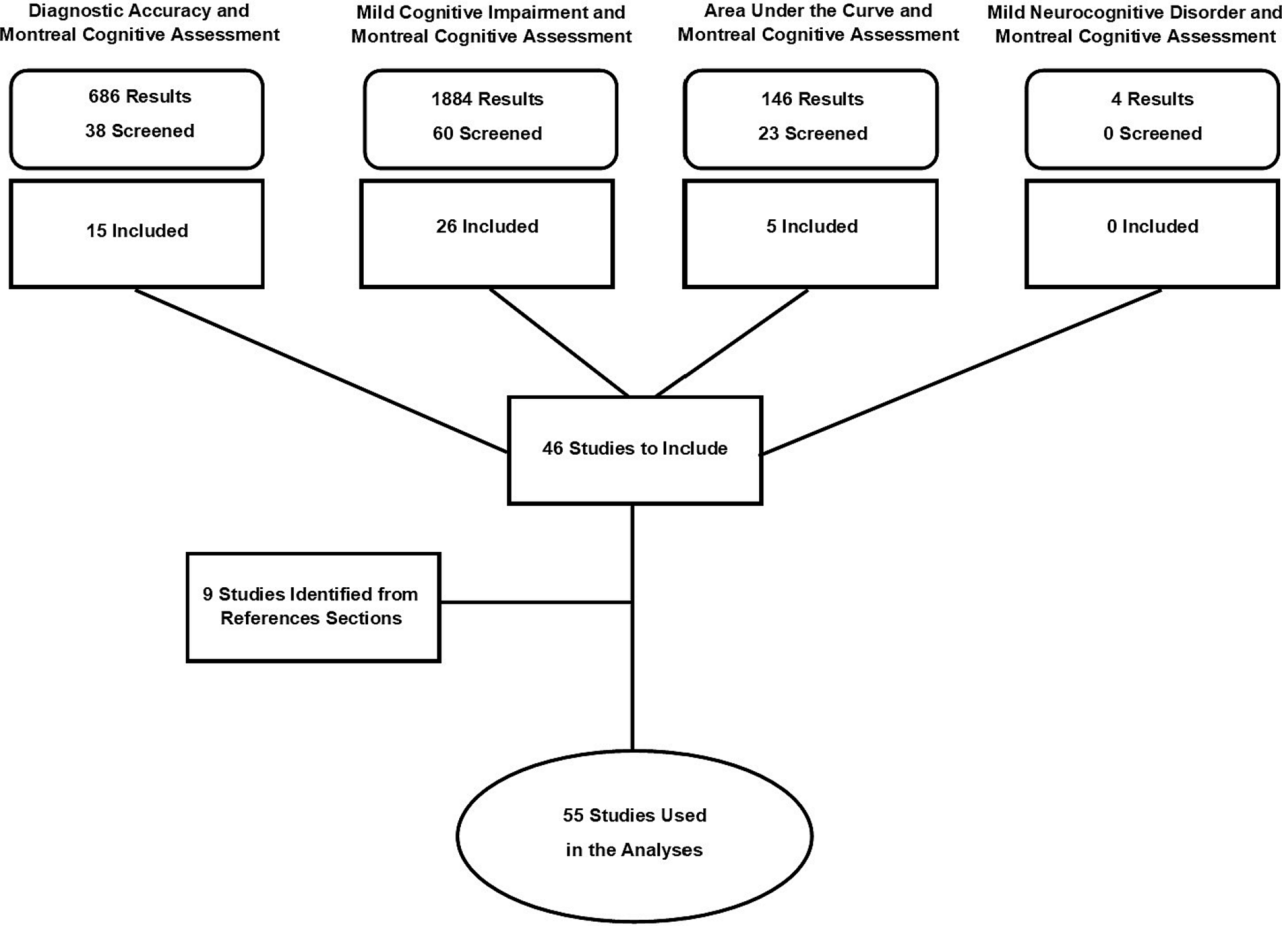
PRISMA flowchart for literature searches and number of studies selected for inclusion.

Prior to conducting the literature searches, the following criteria for study selection and inclusion were established: (1) The data could not come from a treatment or intervention trial, (2) The study should report either raw means and standard deviations for MoCA performance in both the CU and aMCI groups OR the study should report should report area under the curve (AUC) values with standard error (SE) or 95% confidence intervals (CI), (3) The study should use either Petersen criteria (Petersen and Negash, 2008) to classify its aMCI subjects or DSM-V criteria for mild neurocognitive disorder (MND). Although in most circumstances using only one set of diagnostic criteria is preferred, we felt that including studies that used either the Petersen or DSM-V MND criteria would provide greater ecological validity for the study results since the MoCA is used primarily as a screening instrument in general practice settings where formal diagnostic criteria for cognitive impairment are not usually applied. PRISMA guidelines were followed for the analysis and a flow chart depicting study screening and selection is shown in Figure 1.

### Literature search terms

Using the PubMed database, four different search terms were used. The first search term, “diagnostic accuracy and Montreal Cognitive Assessment” yielded 686 results from which 38 were screened and 15 were selected for inclusion. A second search using “mild cognitive impairment and Montreal Cognitive Assessment” yielded 1,884 results from which 60 were screened with 26 that were selected for inclusion. The third search using “area under the curve and Montreal Cognitive Assessment” yielded 146 results with 23 that were selected for screening from which five were included. A fourth search using the term “mild neurocognitive disorder and Montreal Cognitive Assessment” did not yield any additional studies beyond those already identified in the previous searches. All four searches were also carried out in the EMBASE database which yielded no additional articles. Nine additional articles were identified through reviews of references sections of the selected papers which brought the final total of included studies to 55 (Figure 1). The search approach taken for this study is consistent with “a multi-faceted approach that uses a series of searches” as described in the *Cochrane Handbook of Systematic Reviews of Interventions* (Lefebvre et al., 2023).

### Data quality and extraction

From each of the included studies the following data were extracted: sample sizes for the CU and MCI groups, means and standard deviations of MoCA scores for the CU and MCI groups, AUC values with standard errors (SE). When 95% CIs were reported, SE was derived by taking the difference between the AUC estimate and the upper bound of the 95% CI and dividing by 3.92 (Higgins et al., 2022). The cutpoint associated with the AUC estimate, means and standard deviations for age and education levels (when education was reported in years), and the geographic region in which the study was conducted (Asia, Europe, North America) were also extracted from each study. The quality of each study was assessed using the National Heart, Lung, and Blood Institute (NHLBI) Study Quality Assessment of Case Control Studies^1^ which was used to grade each study as Good, Fair, or Poor.

### Statistical analysis

The first analytic approach was a meta-analysis of AUC values derived from the receiver operator characteristic (ROC) analyses that differentiated aMCI from CU individuals. The second analytic approach included analyses of the standardized mean difference (SMD) (Hedge’s *g*) and the raw mean difference (RMD) for MoCA scores between CU and aMCI. For both analytic approaches, results from random effects analyses were reported and the *I*^2^ statistic was used to quantify between-study heterogeneity which was classified as low, moderate, or high based on proposed guidelines (Higgins et al., 2003). Additional AUC and SMD analyses were carried out for subgroups based on geographic region (Asia, Europe, North America). The Egger’s test was used to determine the presence of publication bias among the included studies. In addition, the median of the reported MoCA cutoff score was used to summarize the reported cutoff values for studies in the AUC analysis. Since the included studies came from a number of different geographic regions we anticipated a wide range of reported MoCA cutoff scores so using the median as a summary measure provides an overall estimate of the MoCA’s cutoff that is relatively robust to the variability of reported cutoff values among the studies. All analyses were carried out using MedCalc Statistical Software version 20.109 (MedCalc Software Ltd., Ostend, Belgium,^2^ 2022).

## Results

A total of 55 studies (Fujiwara et al., 2010; Lu et al., 2011; Ahmed et al., 2012; Larner, 2012; Yu et al., 2012; Dong et al., 2013; Freitas et al., 2013; Memória et al., 2013; Roalf et al., 2013; Cummings-Vaughn et al., 2014; Goldstein et al., 2014; Kaya et al., 2014; Malek-Ahmadi et al., 2014; Yeung et al., 2014; Zhou et al., 2014; Chu et al., 2015; Julayanont et al., 2015; Ng et al., 2015; Trzepacz et al., 2015; Mellor et al., 2016; O’Caoimh et al., 2016; Tsai et al., 2016; Cecato et al., 2017; Clarnette et al., 2017; Janelidze et al., 2017; Bartos and Fayette, 2018; Chiu et al., 2018; Lee et al., 2018; Li et al., 2018; Cesar et al., 2019; Delgado et al., 2019; Rossetti et al., 2019; Townley et al., 2019; Wang et al., 2019; Pinto et al., 2019b; Aycicek et al., 2020; Bello-Lepe et al., 2020; Dautzenberg et al., 2020; Freud et al., 2020; Senda et al., 2020; Serrano et al., 2020; Sokołowska et al., 2020; Thomann et al., 2020; González et al., 2021; Hemrungrojn et al., 2021; Masika et al., 2021; Rashedi et al., 2021; Rodríguez-Salgado et al., 2021; Yan et al., 2021; Pan et al., 2022; Paterson et al., 2022) were included in this meta-analysis from which 40 were used in the AUC analysis and 45 were used in the analysis of mean differences. Thirty-one of the included studies were used in both the AUC and mean difference analyses. An AUC-derived MoCA cutoff score for aMCI was reported by 45 studies. There was a great deal of diversity in the language of administration among the included studies which is shown in Table 1. English was the most prevalent among the studies (*n* = 15) followed by Mandarin (*n* = 11), Portuguese (*n* = 6), and Spanish (*n* = 4). 41% of the included studies were judged to be of good quality while 59% were judged to be of fair quality.

**Table 1:** Distribution of MoCA administration language among included studies.

**Language of administration**	**Number of studies**
English	15
Mandarin	11
Portuguese	6
Spanish	4
Cantonese	3
Japanese, Turkish	2
Czech, Dutch, Farsi, Georgian, German, Hebrew, Kiswahili, Malay, Mandarin and Malay, Polish, Russian, Thai	1

**Figure 2:**
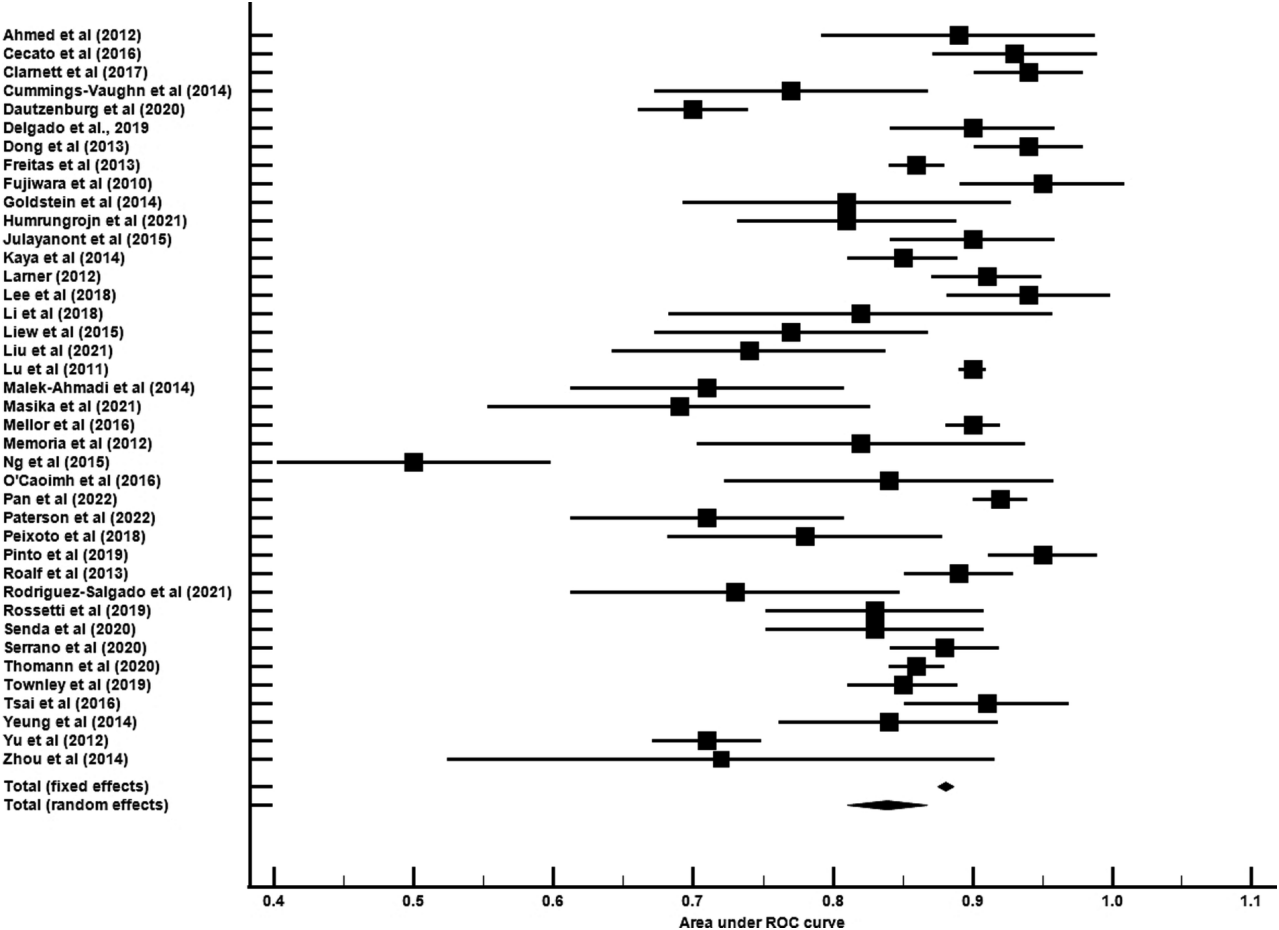
Forest plot of MoCA diagnostic accuracy for amnestic mild cognitive impairment.

**Figure 3:**
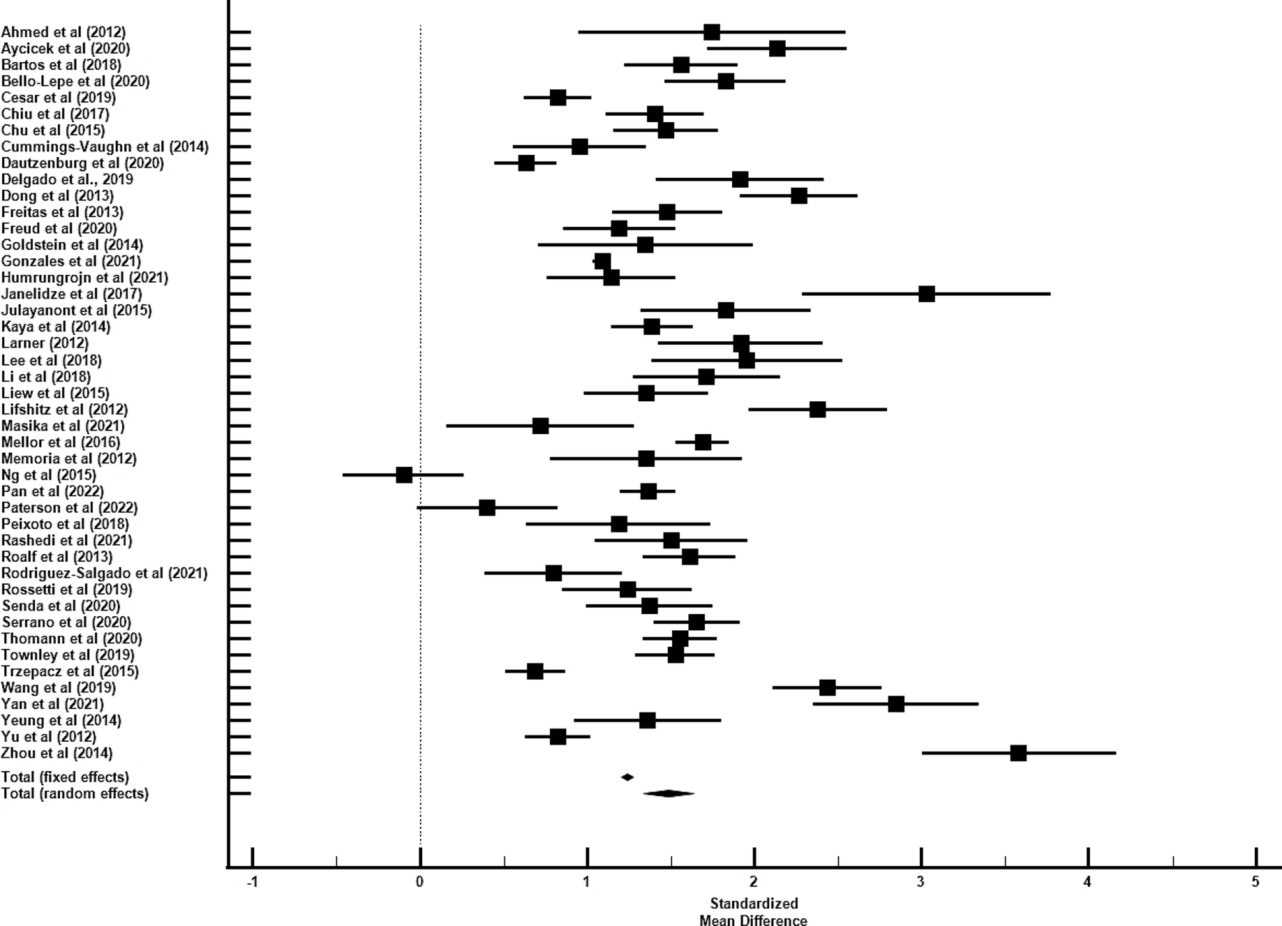
Forest plot for standardized mean difference of MoCA performance between CU and aMCI groups.

**Figure 4:**
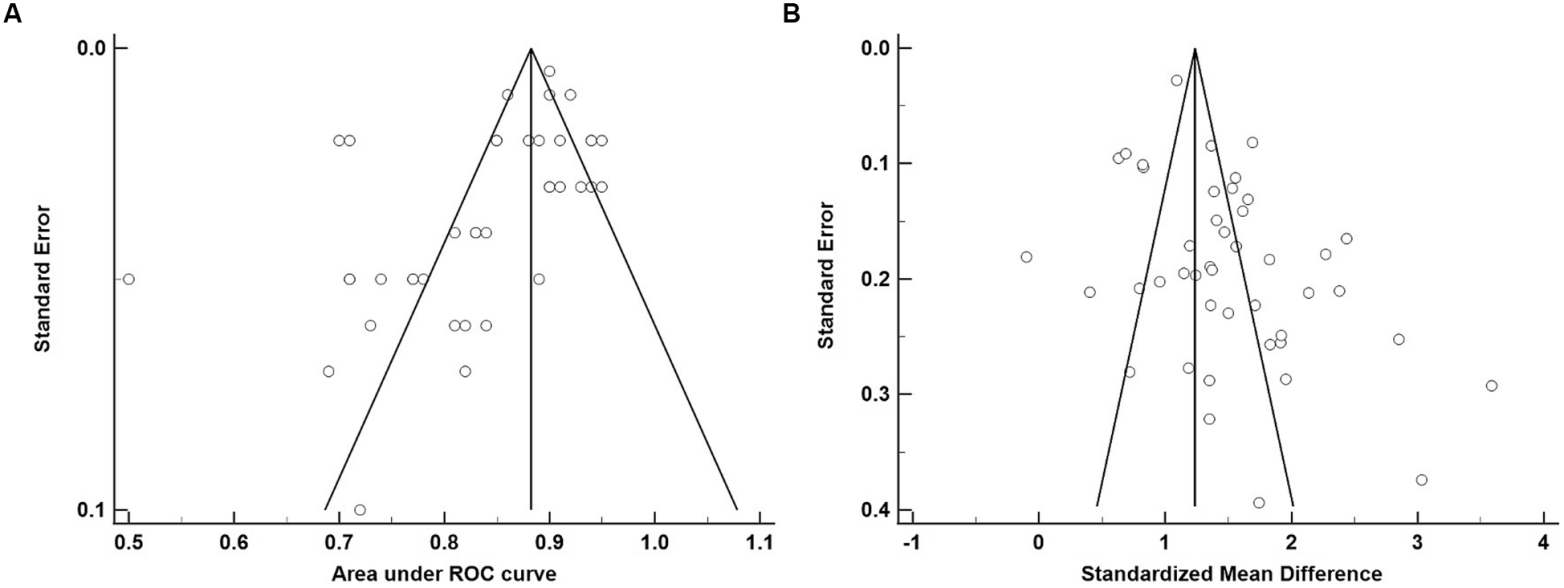
Funnel plots for MoCA diagnostic accuracy **(A)** and standardized mean difference **(B)**.

The average age for CU groups was 71.06 ± 7.37 years with an average of 11.44 ± 3.27 years of education. For aMCI groups, the average age was 73.99 ± 7.65 years with an average of 9.89 ± 3.47 years of education. Mean MoCA scores for the CU groups was 24.98 ± 2.88 and 20.11 ± 3.76 for the aMCI groups. Among studies that reported optimal cutoff values (*n* = 44), the median was 24 (range = 17–27). Characteristics of each study included in the AUC meta-analysis are shown in Table 2. The overall AUC value was 0.84, 95% CI (0.81, 0.87), *p* < 0.001 with very high heterogeneity [*I*^2^ = 90, 95% CI (87, 92%)] (Figure 2). The Egger’s test indicated the presence of publication bias in the analysis (*p* = 0.002). The meta-analysis for differences in means demonstrated a large effect size [Hedge’s *g* = 1.49, 95% CI (1.33, 1.64), *p* < 0.001; Table 3] with very high heterogeneity [*I*^2^ = 93, 95% CI (91, 94%)] and an Egger’s test that indicated the presence of publication bias (*p* = 0.003). The large effect size reported here equates to a 4.73 (95% CI: 4.20, 5.27) point difference on the MoCA between CU and aMCI groups (Figure 3). Characteristics of each study included in the SMD meta-analysis are shown in Table 2. Funnel plots depicting the publication bias in the AUC and SMD analyses are shown in Figure 4.

**Table 2:** Characteristics of studies used in the diagnostic accuracy meta-analysis.

**Study**	**CU sample size**	**CU age**	**aMCI sample size**	**aMCI age**	**AUC ± SE**	**Cutoff score**
Ahmed et al. (2012)	20	77.4 ± 4.0	15	80.9 ± 7.2	0.89 ± 0.05	23
Cecato et al. (2017)	39	71.6 ± 6.9	44	76.7 ± 7.0	0.93 ± 0.03	24
Clarnette et al. (2017)	41	nr	72	nr	0.94 ± 0.02	23
Cummings-Vaughn et al. (2014)	51	77 ± 7.5	57	78.8 ± 6.7	0.77 ± 0.05	24
Dautzenberg et al. (2020)	459	71.3 ± 7.3	153	73.9 ± 8	0.70 ± 0.02	21
Delgado et al. (2019)	104	72.3 ± 5.4	24	75.3 ± 7.8	0.90 ± 0.03	21
Dong et al. (2013)	128	67.4 ± 4.8	83	74.3 ± 5.5	0.94 ± 0.02	24
Freitas et al. (2013)	90	69.6 ± 7.1	90	70.5 ± 8.0	0.86 ± 0.01	22
Fujiwara et al. (2010)	36	76.4 ± 3.3	30	77.3 ± 6.3	0.95 ± 0.03	25
Goldstein et al. (2014)	16	65.8 ± 7.7	38	71.9 ± 8.9	0.81 ± 0.06	24
Hemrungrojn et al. (2021)	60	67.9 ± 6.4	61	72.1 ± 7.0	0.81 ± 0.04	24
Julayanont et al. (2015)	43	66.6 ± 6.7	42	70.2 ± 6.6	0.90 ± 0.03	25
Kaya et al. (2014)	246	68.0 ± 10.3	114	74.2 ± 8.8	0.85 ± 0.02	nr
Larner (2012)	85	nr	29	nr	0.91 ± 0.02	20
Lee et al. (2018)	35	73.6 ± 6.4	36	76.2 ± 7.4	0.94 ± 0.03	nr
Li et al. (2018)	53	70.2 ± 9.1	56	75.2 ± 7.1	0.82 ± 0.07	24
Liew et al. (2015)	146	64.9 ± 7.0	41	71.8 ± 6.7	0.77 ± 0.05	25
Liu et al. (2021)	50	68.0 ± 8.2	50	76.7 ± 10.8	0.74 ± 0.05	23
Lu et al. (2011)	6,283	72.0	1,687	75.1	0.90 ± 0.005	25
Malek-Ahmadi et al. (2014)	73	82.6 ± 7.7	39	80.5 ± 8.4	0.71 ± 0.05	nr
Masika et al. (2021)	19	69.3 ± 5.8	42	70.4 ± 8.0	0.69 ± 0.07	19
Mellor et al. (2016)	708	72.5 ± 8.4	267	76.5 ± 7.7	0.90 ± 0.01	24
Memória et al. (2013)	28	72.5 ± 5.3	30	74.7 ± 5.7	0.82 ± 0.06	nr
Ng et al. (2015)	88	nr	46	nr	0.50 ± 0.05	nr
O’Caoimh et al. (2016)	101	nr	103	nr	0.84 ± 0.06	24
Pan et al. (2022)	431	66.5 ± 9.3	285	72.1 ± 10.5	0.92 ± 0.01	23
Paterson et al. (2022)	40	74.0 ± 7.0	51	75.0 ± 5.7	0.71 ± 0.05	nr
Peixoto et al. (2018)	30	68.6 ± 6.2	30	67.2 ± 9.3	0.78 ± 0.05	22
Pinto et al. (2019a,b)	110	nr	88	nr	0.95 ± 0.02	nr
Roalf et al. (2013)	140	71.2 ± 9.2	126	72.3 ± 8.1	0.73 ± 0.06	nr
Rodríguez-Salgado et al. (2021)	53	70.4 ± 5.9	46	72.7 ± 7.5	0.73 ± 0.06	nr
Rossetti et al. (2019)	45	62.3 ± 6.8	90	64.8 ± 5.9	0.83 ± 0.04	24
Senda et al. (2020)	50	64.9 ± 12.0	94	73.5 ± 8.3	0.83 ± 0.04	nr
Serrano et al. (2020)	155	71.5 ± 6.2	158	72.6 ± 6.3	0.88 ± 0.02	25
Thomann et al. (2020)	283	73.8 ± 5.2	159	76.0 ± 6.0	0.86 ± 0.01	25
Townley et al. (2019)	313	81.7 ± 5.0	114	84 ± 5.2	0.85 ± 0.02	24
Tsai et al. (2016)	26	nr	59	nr	0.91 ± 0.03	27
Yeung et al. (2014)	49	73.6 ± 7.6	49	76.5 ± 7.5	0.84 ± 0.04	21
Yu et al. (2012)	865	70.4 ± 7.1	115	71.5 ± 7.3	0.71 ± 0.02	21
Zhou et al. (2014)	148	67.7 ± 7.2	24	67.2 ± 6.6	0.72 ± 0.10	26

CU, cognitively unimpaired; MCI, mild cognitive impairment; AUC, area under the curve; SE, standard error; nr, not reported.

**Table 3:** Characteristics of studies used in the standardized mean difference meta-analysis.

	**Cognitively unimpaired**			**Mild cognitive impairment**		
**Study**	**Sample size**	**Age**	**MoCA**	**Sample size**	**Age**	**MoCA**
Ahmed et al. (2012)	20	77.4 ± 4.0	27.1 ± 2.8	15	80.9 ± 7.2	21.7 ± 3.3
Aycicek et al. (2020)	91	71.0	22.8 ± 3.2	54	75.0	14.2 ± 5.1
Bartos and Fayette (2018)	226	72.0 ± 8.0	26.0 ± 3.0	48	72.0 ± 7.0	21.0 ± 4.0
Bello-Lepe et al. (2020)	113	71.5 ± 7.6	23.7 ± 3.2	65	76.92 ± 8.71	17.2 ± 4.1
Cesar et al. (2019)	385	nr	19.1 ± 4.9	135	nr	15.1 ± 4.6
Chiu et al. (2018)	99	75.4 ± 6.6	23.1 ± 3.4	128	76.4 ± 6.8	18.3 ± 3.4
Chu et al. (2015)	115	72.2 ± 6.1	24.4 ± 3.2	87	77.2 ± 6.3	18.7 ± 4.6
Cummings-Vaughn et al. (2014)	51	77 ± 7.5	25.8 ± 2.9	57	78.8 ± 6.7	22.8 ± 3.3
Dautzenberg et al. (2020)	459	71.3 ± 7.3	23.5 ± 4.2	153	73.9 ± 8	20.9 ± 3.8
Delgado et al. (2019)	104	72.3 ± 5.4	24.2 ± 3.7	24	75.3 ± 7.8	17.0 ± 3.9
Dong et al. (2013)	128	67.4 ± 4.8	24.3 ± 2.8	83	74.3 ± 5.5	16.4 ± 4.3
Freitas et al. (2013)	90	69.6 ± 7.1	23.6 ± 3.2	90	70.5 ± 8.0	18.3 ± 3.9
Freud et al. (2020)	80	80.1 ± 7.1	24.3 ± 3.7	80	75.0 ± 5.3	20.2 ± 3.1
Goldstein et al. (2014)	16	65.8 ± 7.7	25.1 ± 2.9	38	71.9 ± 8.9	19.8 ± 4.2
González et al. (2021)	3,905	68.0 ± 10.4	26.0 ± 3.0	2,362	70.4 ± 9.0	22.0 ± 4.6
Hemrungrojn et al. (2021)	60	67.9 ± 6.4	28.5 ± 1.8	61	72.1 ± 7.0	26.2 ± 2.2
Janelidze et al. (2017)	46	57.7 ± 10.8	26.3 ± 2.5	20	62.8 ± 11.5	19.2 ± 1.8
Julayanont et al. (2015)	43	66.6 ± 6.7	26.6 ± 1.9	42	70.2 ± 6.6	22.9 ± 2.1
Kaya et al. (2014)	246	68.0 ± 10.3	23.3 ± 3.1	114	74.2 ± 8.8	18.9 ± 3.3
Larner (2012)	85	nr	25.2 ± 3.2	29	nr	18.3 ± 4.5
Lee et al. (2018)	35	73.6 ± 6.4	24.5 ± 2.5	36	76.2 ± 7.4	16.6 ± 5.1
Li et al. (2018)	53	70.2 ± 9.1	25.8 ± 2.3	56	75.2 ± 7.1	20.9 ± 3.3
Liew et al. (2015)	146	64.9 ± 7.0	25.2 ± 2.1	41	71.8 ± 6.7	21.6 ± 4.0
Lifshitz et al. (2012)	80	71.3 ± 4.7	26.7 ± 1.9	74	76.3 ± 5.6	20.3 ± 3.3
Masika et al. (2021)	19	69.3 ± 5.8	20.1 ± 5.4	42	70.4 ± 8.0	15.9 ± 5.9
Mellor et al. (2016)	708	72.5 ± 8.4	27.6 ± 2.7	267	76.5 ± 7.7	21.4 ± 5.5
Memória et al. (2013)	28	72.5 ± 5.3	26.3 ± 2.9	30	74.7 ± 5.7	22.1 ± 3.3
Ng et al. (2015)	88	nr	26.5 ± 3.2	46	nr	26.8 ± 2.7
Pan et al. (2022)	431	66.5 ± 9.3	26.3 ± 3.5	285	72.1 ± 10.5	20.5 ± 5.1
Paterson et al. (2022)	40	74.0 ± 7.0	25.0 ± 2.3	51	75.0 ± 5.7	24.0 ± 2.6
Peixoto et al. (2018)	30	68.6 ± 6.2	26.3 ± 2.5	30	67.2 ± 9.3	21.6 ± 4.9
Rashedi et al. (2021)	59	62.6 ± 6.7	24.5 ± 3.0	40	68.1 ± 8.8	19.3 ± 4.0
Roalf et al. (2013)	140	71.2 ± 9.2	26.8 ± 2.6	126	72.3 ± 8.1	20.9 ± 4.5
Rodríguez-Salgado et al. (2021)	53	70.4 ± 5.9	27.1 ± 2.2	46	72.7 ± 7.5	25.3 ± 2.3
Rossetti et al. (2019)	45	62.3 ± 6.8	25.5 ± 2.1	90	64.8 ± 5.9	21.3 ± 3.9
Senda et al. (2020)	50	64.9 ± 12.0	25.6 ± 2.7	94	73.5 ± 8.3	21.6 ± 3.0
Serrano et al. (2020)	155	71.5 ± 6.2	25.5 ± 2.2	158	72.6 ± 6.3	20.6 ± 3.5
Sokołowska et al. (2020)*	91	74.1	25.9	190	78.2	21.82
Thomann et al. (2020)	283	73.8 ± 5.2	26.5 ± 2.4	159	76.0 ± 6.0	22.0 ± 3.6
Townley et al. (2019)	313	81.7 ± 5.0	24.5 ± 2.5	114	84.0 ± 5.2	20.5 ± 2.9
Trzepacz et al. (2015)	219	77.7 ± 6.2	25.6 ± 2.8	299	74.2 ± 7.9	23.4 ± 3.4
Wang et al. (2019)	136	69.2 ± 11.4	26.5 ± 2.1	120	76.9 ± 7.9	20.2 ± 3.1
Yan et al. (2021)	64	73.5 ± 16.0	26.6 ± 1.0	62	82.0 ± 15.5	20.8 ± 2.7
Yeung et al. (2014)	49	73.6 ± 7.6	22.6 ± 4.0	49	76.5 ± 7.5	16.4 ± 5.0
Yu et al. (2012)	865	70.4 ± 7.11	22.3 ± 5.4	115	71.5 ± 7.3	17.8 ± 6.3
Zhou et al. (2014)	148	67.7 ± 7.2	21.5 ± 0.7	24	67.2 ± 6.6	18.3 ± 1.6

Mean ± standard deviation; nr, not reported. ^*^Standard deviation was not reported.

Analyses of ROC values by geographic region (Table 4) found that North American and Asian studies both yielded AUC values of 0.84 with European studies having a slightly higher AUC of 0.85. For the region-wise SMD analysis (Table 4), Asian studies had the largest effect size [Hedge’s *g* = 1.67, 95% CI (1.33, 2.01)], followed by North America [Hedge’s *g* = 1.23, 95% CI (1.05, 1.49)] and Europe [Hedge’s g = 1.21, 95% CI (0.87, 1.56)].

**Table 4:** Diagnostic accuracy and standardized mean difference analyses stratified by global region.

**Global region**	**AUC (95% CI)**	***p*-value**	***I*** ^**2**^ **(95% CI)**
North America	0.84 (0.80, 0.88)	<0.001	78% (62, 87%)
Asia	0.84 (0.78, 0.89)	<0.001	93% (89, 95%)
Europe	0.85 (0.79, 0.90)	<0.001	92% (87, 95%)

**Global region**	**Hedge’s *g* (95% CI)**	***p*-value**	***I*** ^**2**^ **(95% CI)**
North America	1.23 (1.05, 1.49)	<0.001	87% (78, 92%)
Asia	1.67 (1.33, 2.01)	<0.001	95% (93, 96%)
Europe	1.21 (0.87, 1.56)	<0.001	90% (82, 95%)

## Discussion

This meta-analysis assessed the diagnostic accuracy and the mean difference of the MoCA when comparing aMCI older adults to those who are CU across several global regions. The overall AUC value of 0.84 indicates that the MoCA has good diagnostic accuracy for aMCI, however a very high degree of between-study heterogeneity was noted for this finding. The analysis of MoCA mean differences yielded a large effect size (Hedge’s *g* = 1.49) and a high degree of between-study heterogeneity was also noted for this analysis. English and Mandarin studies (*n* = 26) made up approximately half of the studies included in the meta-analysis and among the studies that assessed diagnostic accuracy a score of 24 was the most commonly-used cutoff for differentiating aMCI from CU individuals. However, it was noted that the range of reported cutoff values was 17 to 27 which suggests that optimal MoCA cutpoints may be population- and context-specific in order to avoid misclassification errors. A recent systematic review highlights this point by noting that cross-cultural differences necessitate the use of varying cutoff values as well as corrections for educational levels in different populations (O’Driscoll and Shaikh, 2017).

Others have also noted significant problems with misclassification on the MoCA when a single cutoff is used as higher rates of false positive indications of impairment were noted with increased age and decreased educational levels (Wong et al., 2015). Based on these previous reports the high levels of between-study heterogeneity in this meta-analysis may reflect the cultural, linguistic, and educational diversity among the included studies rather than any particular methodological weakness among them. These findings also emphasize the need to frame the MoCA’s utilization in a screening rather than a diagnostic context. Here it is also important to consider the sensitivity and specificity of a cognitive screening measure and how this impacts the utilization of full neuropsychological evaluations. The high false-positive rates of impairment on the MoCA using 26 as the cutoff could lead to many CU individuals being referred for unnecessary neuropsychological evaluations (Ilardi et al., 2023). In contrast, lowering the cutoff score for impairment also has the effect reducing the MoCA’s sensitivity in correctly detecting aMCI which further underscores the notion that the MoCA’s cutoff score can be adjusted for a given population in order to optimize its diagnostic accuracy. Additionally, adjustments to the cutoff score can be made when physical limitations (e.g., hearing loss) substantially impact MoCA performance (Utoomprurkporn et al., 2020).

A previous meta-analysis of nine studies investigating the MoCA’s diagnostic accuracy showed that the optimal MoCA cutoff for detecting aMCI was 23 (Carson et al., 2018) and a recent systematic review found that the AUC value for the MoCA in differentiating aMCI from CU individuals ranged from 0.71 to 0.99 across 34 studies (Pinto et al., 2019b) putting the AUC value of this meta-analysis (0.84) near the midpoint of this range. The three global regions examined in this meta-analysis (North America, Asia, Europe) all had comparable AUC and effect size values despite each region having a high degree of between-study heterogeneity.

There are some limitations to this meta-analysis. Despite the very large number of studies included for both the AUC and SMD analyses, a high degree of between-study heterogeneity was noted for all analyses which decreases the level of confidence one may have in the findings that are reported. A number of different factors may account for the high heterogeneity such as study setting (clinic vs. community-based), varying educational attainment of the populations among the different geographic regions, and cultural norms and values that may impact test performance. While the inconsistencies of the reported AUC and SMD values across studies warrant some degree of skepticism for the final results, there is also significant value in findings that are derived from such a large number of studies across different geographic regions and this aspect of the meta-analysis will likely appeal to clinicians who use the MoCA.

The findings of this meta-analysis provide further support for the use of the MoCA as an accurate cognitive screening tool for use in general practice settings. In line with other studies of the MoCA in aMCI and CU samples, a score of 24 appears to be the optimal cutoff to use for identifying cognitive impairment.

## Data availability statement

The original contributions presented in the study are included in the article/Supplementary material, further inquiries can be directed to the corresponding author.

## Author contributions

MM-A: Conceptualization, Data curation, Formal analysis, Methodology, Supervision, Writing – original draft, Writing – review & editing. NN: Data curation, Formal analysis, Writing – original draft, Writing – review & editing.

## Footnotes

^1^ https://www.nhlbi.nih.gov/health-topics/study-quality-assessment-tools

^2^ https://www.medcalc.org
